# Supplementary material for: Interactome disassembly during apoptosis occurs independent of caspase cleavage
Source: Mol Syst Biol. 2017 Jan 12;13(1):906. doi: 10.15252/msb.20167067 (PMC5293159; doi:10.15252/msb.20167067)
Supplement: Supplementary file 1 — Appendix [file MSB-13-906-s001.pdf]

Interactome disassembly during apoptosis occurs independent of caspase cleavage

Nichollas E. Scott[1,2], Lindsay D. Rogers[3,4], Anna Prudova[1,2], Nat F. Brown[1,2], Nikolaus Fortelny[1,2,4], Christopher M. Overall[2,3,4], and Leonard J. Foster[1,2]

[1]Centre for High-Throughput Biology, University of British Columbia, Vancouver, British Columbia, Canada, V6T 1Z4.

[2]Department of Biochemistry and Molecular Biology, University of British Columbia, Vancouver, British Columbia, Canada.

[3]Department of Oral Biological and Medical Sciences, University of British Columbia, Vancouver, British Columbia, Canada.

[4]Centre for Blood Research, University of British Columbia, Vancouver, British Columbia, Canada.

Appendix Table of Contents

Titles	Page number
Experimental Design Description	Page 2
Figure S1, Establishment of the BN-PCP-SILAC approach	Page 3
Figure S2, BN-PCP-SILAC SEC coverage and quantitation	Page 4
Figure S3, Markov clustering of binary interactions determined from PCP-BN-SILAC	Page 5
Figure S4, Fas-mediated initiation of apoptosis time course	Page 6
Figure S5, Vertex degree distribution of interactomes	Page 7
Figure S6, Distribution of Markov clustered complexes within interactomes	Page 8
Figure S7, Fas-mediated changes in the cytoplasmic and membrane interactomes at 1 hour post treatment	Page 9
Figure S8, tBID BN-PCP-SILAC protein and peptide profiles	Page 10
Figure S9, Changes in the protein and peptide profiles of nuclei related components observed in the cytoplasmic interactome	Page 11
Figure S10, Analysis of Z-vad FMK stabilized N-termini	Page 12
Figure S11, Investigation of alterative protease activities in response to Fas initiated apoptosis	Page 13
Figure S12, Map of Filamin-B (O75369)	Page 14
Figure S13, Investigation of protein specific proteolysis of known caspase targets	Page 15
Figure S14, BN-PCP-SILAC Protein and Peptide Profiles of the known caspae-3 target NADH dehydrogenase (P28331)	Page 16
Datasets descriptions	Page 17
Appendix References	Page 18

Experimental Design:

Fas-mediated apoptosis leading to caspase activation is well characterised within Jurkat cells (Scaffidi et al, 1998; Van Damme et al, 2005). Within this cell type the loss of mitochondria transmembrane potential and the release of cytochrome C via the insertion of tBID into the mitochondria (Li et al, 1998) are required for the successful activation of executioner caspases (Scaffidi et al, 1998). This activation occurs within 4 hours of Fas treatment and progressively amplifies committing the cell to destruction (Scaffidi et al, 1998; Van Damme et al, 2005; Weis et al, 1995). However, the precise roles of caspase cleavage events are uncertain—do they disassemble essential protein complexes and cellular machinery by selective cleavage of key proteins, or does unrelenting cleavage eventually exhaust cellular resources leading to inevitable cell death? To explore the temporal and spatial link between proteolytic processing and protein:protein interactions both high content interactome proteomics and high fidelity N-termini proteomic identification of proteins in complexes were performed (Figure S1). To determine caspase-specific cleavages versus cleavages from other protease classes, TAILS was performed with and without caspase inhibition using the inhibitor Z-vad-FMK. As the release of cytochrome C is dependent on the remodelling of the mitochondrial membrane and is a critical step in the initiation of apoptosis comprehensive analysis of interaction changes requires characterisation of both organelle and cytosolic interactomes. To achieve this we established a protein correlation profiling (PCP) strategy compatible with the characterisation of organelle interactomes (Figure S2) and complemented this analysis with our established size exclusion chromatography (SEC) approach (Kristensen et al, 2012). Combining these two powerful approaches enabled the characterisation of protein alterations specifically associated with the initiation of apoptosis. Finally, we further deepened our interrogation of these proteins and their interaction complexes by identifying proteolytic processing events that were caspase dependent and caspase independent using TAILS.

Supplementary Figure legend

Scott N E et al 2016

Figure S1, Workflow of the BN-PCP-SILAC approach

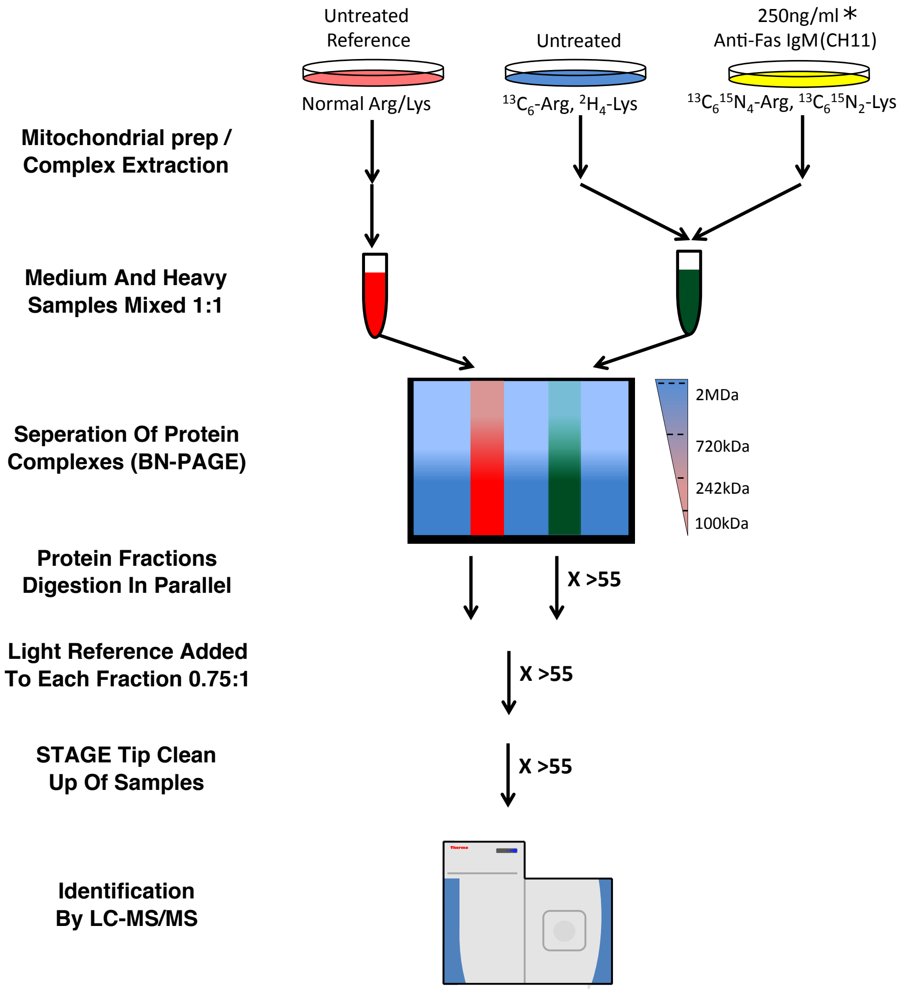

* For initial optimisation experiments no treatment was used

Figure S1, Establishment of the BN-PCP-SILAC approach: A diagrammatic workflow of the BN-PAGE approach: samples are labelled using SILAC and isolated in parallel. Prior to BN separation, isotopically labelled samples (denoted by the yellow and blue samples) are combined (forming the green sample) and separated together. Gel slices are generated and in-gel digestion performed. The digested reference isotopologue labelled samples, shown in red, is then aliquoted into each gel fraction digests and sample cleaned up using C18 enrichment followed by LC-MS analysis.

Figure S2A, Protein Coverage Across BN-PAGE Fractionations

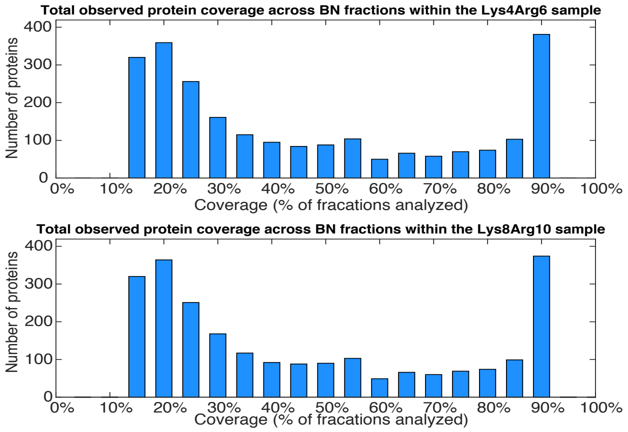

Figure S2B, Protein Overlap With Previous BN-PAGE Studies

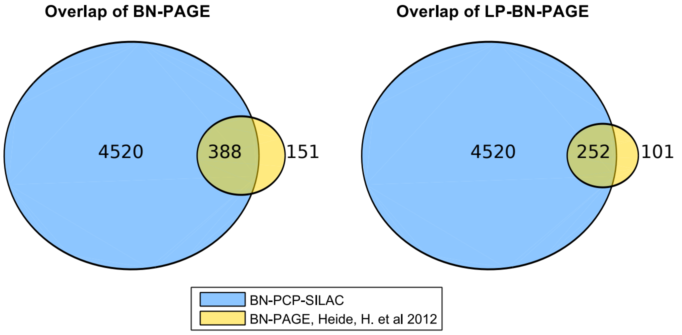

Figure S2C, Protein Group Quantifiable Within Both Isotopolgue Channels

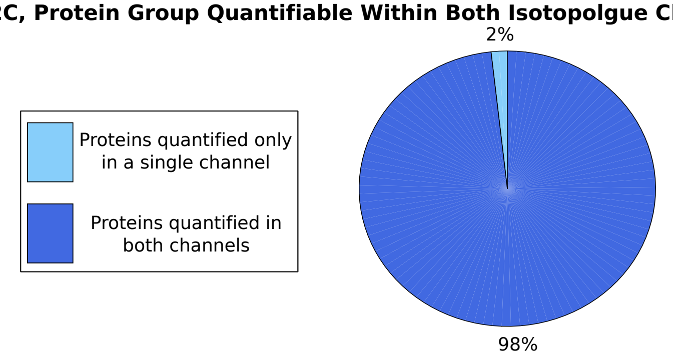

Figure S2, BN-PCP-SILAC SEC coverage and quantitation: A) Protein coverage observed across BN-PCP-SILAC fractionation. Using BN-PAGE separation, the majority of proteins were quantified in >50% of SEC fractions, enabling the generation of robust protein profiles in both isotopologue channels. **B)** Comparison of overlap in protein groups observed using BN page and large pore (LP) BN Page by Heide *et al* (Heide et al, 2012) and this study. **C)** Pie chart of quantitation of observed protein groups where across isotopologue channels. Group one represents the percentage of proteins observed in only a single isotopologue channel and Group two represents the percentage of proteins observed in both isotopologue channels. Of the quantified proteins 98% of proteins were quantified in both isotopologue channels.

Figure S3, Markov Clustering Of Binary Interactions Determined From PCP-BN-SILAC

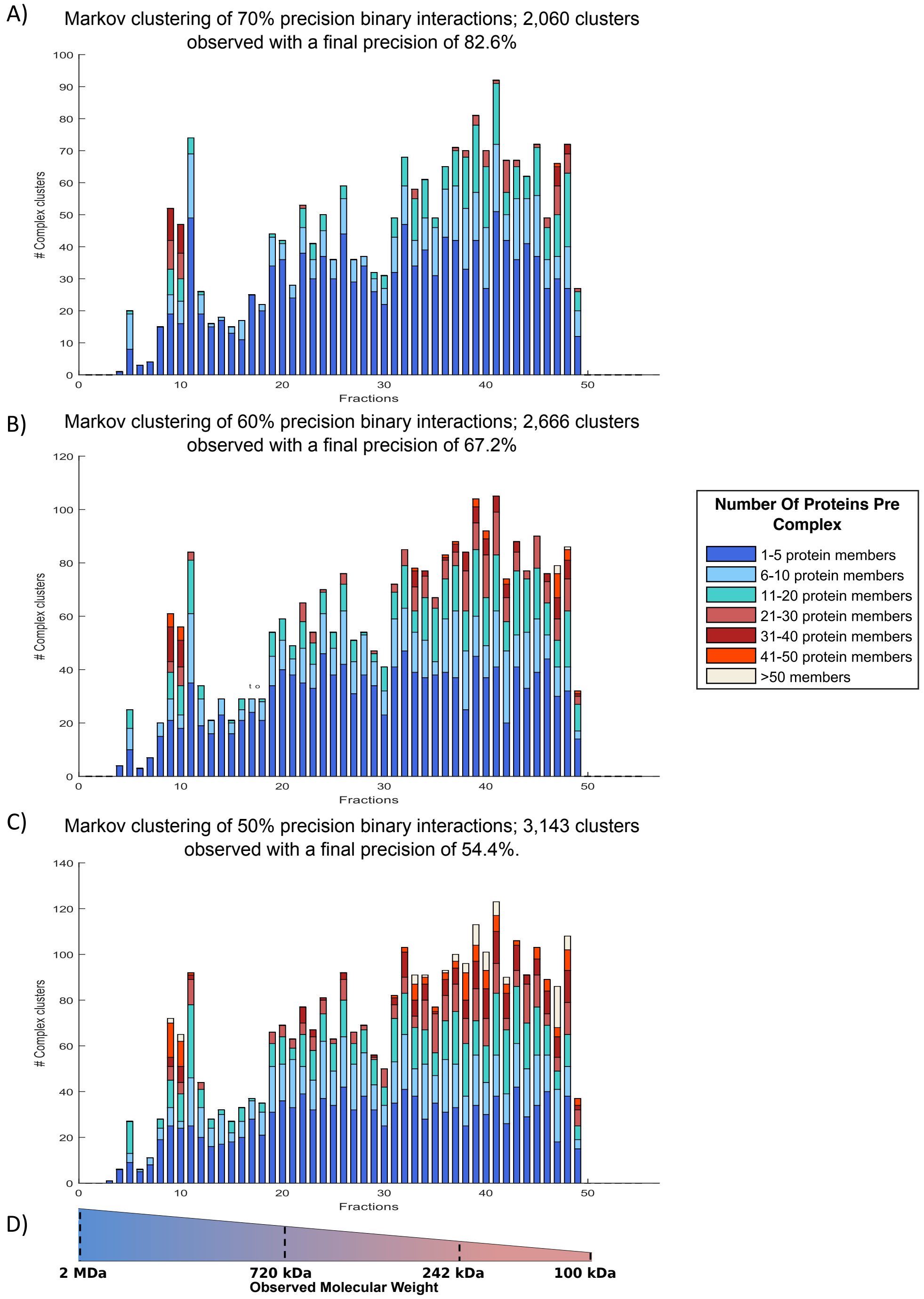

Figure S3, Markov clustering of binary interactions determined from PCP-BN-SILAC: Complexes observed within the PCP-BN-SILAC interactome based on Markov clustering mapped to position within the separation gradients. **A)** 70% precision, 2060 clusters with a final precision of 82.6%, **B)** 60% precision, 2666 clusters with a final precision of 67.2%, **C)** 50% precision, 3143 clusters, with a final precision of 54.4%. **D)** Molecular weight ruler of BN-PAGE based on NativeMark stained Protein Standard.

Figure S4, Fas-Induced Initiation Of Apoptosis Time Course

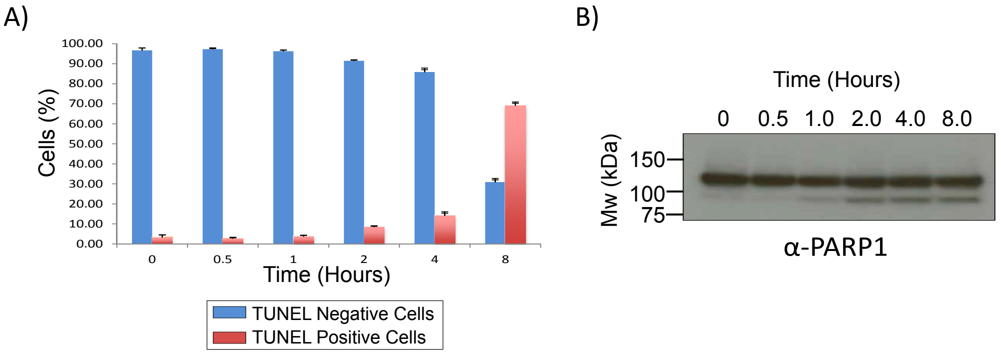

Figure S4, Fas-mediated initiation of apoptosis time course: A) FAC analysis of Fas-mediated apoptosis time course. The addition of anti-Fas IgM antibody (CH11) leads to an increase in the proportion of TUNEL positive cells during the initiation of apoptosis. **B)** Western analysis of poly(ADP-ribose) polymers (*PARP*1) during the initiation of apoptosis. During the initiation of apoptosis increasing amounts of cleaved PARP-1 are generated due to the activation of caspase activity.

Figure S5, Vertex Degree Distribution Of Interactomes

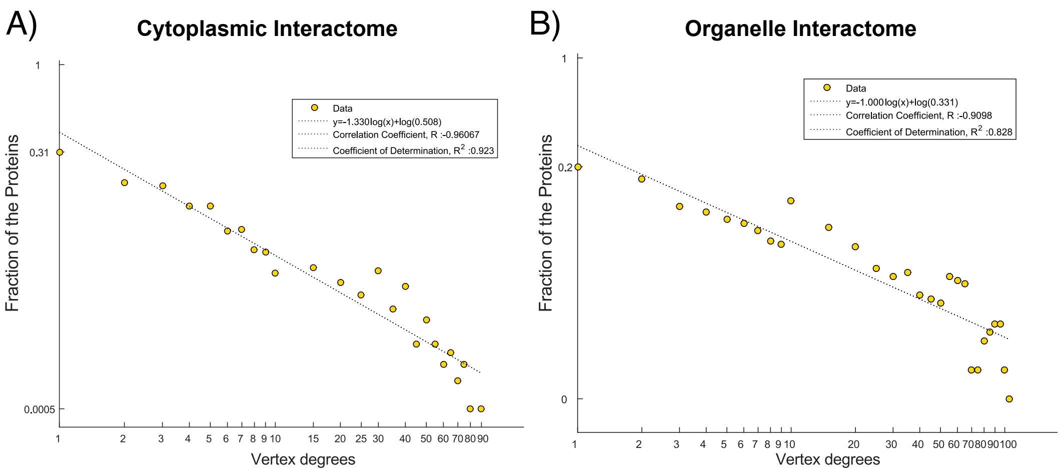

Figure S5, Vertex degree distribution of interactomes: A) Cytoplasmic interactome (determined at a 70% precision), **B)** Organelle interactome (determined at a 70% precision).

Figure S6, Distribution Of Markov Clustered Complexes Within Interactomes

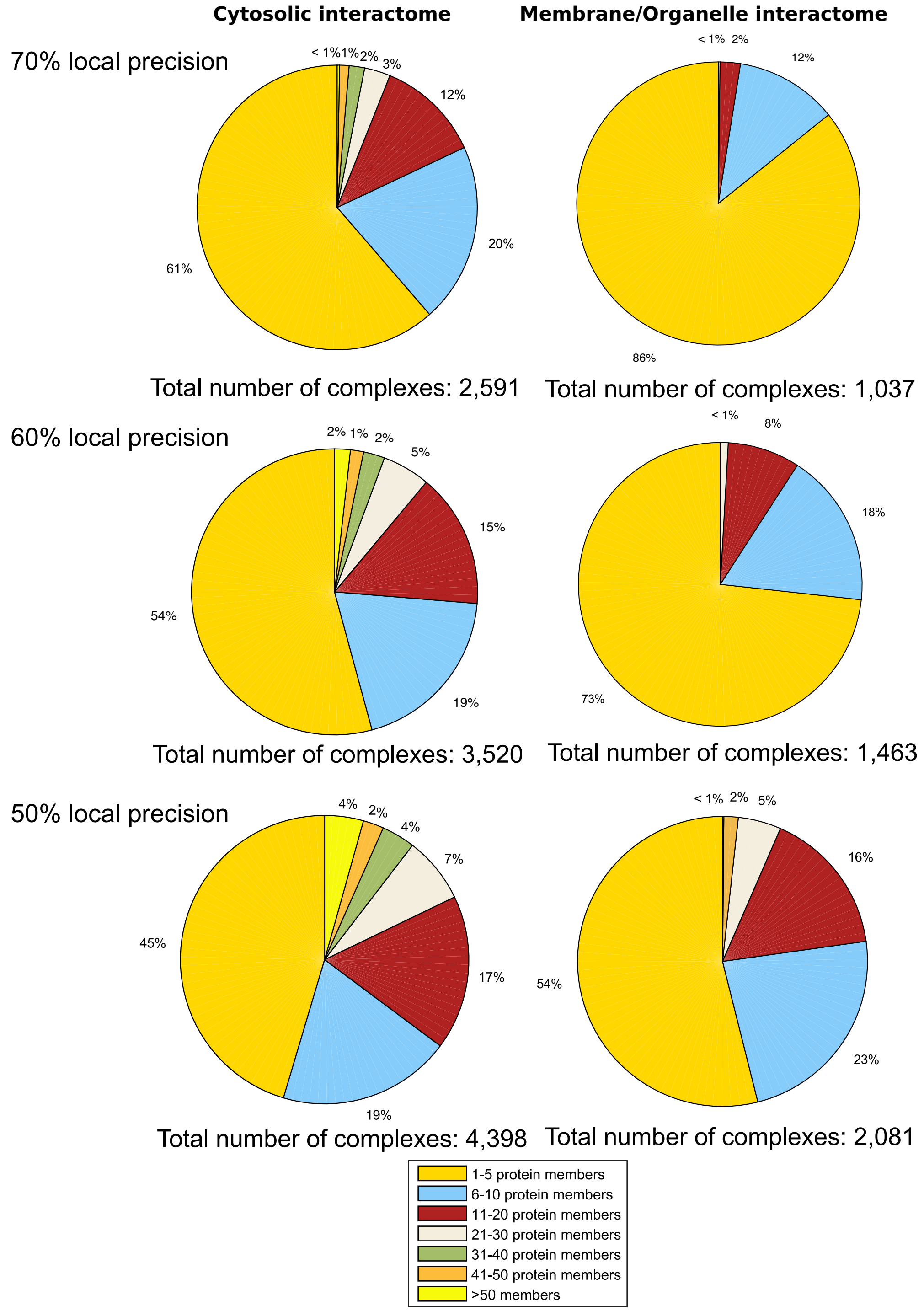

Figure S6, Distribution of Markov clustered complexes within interactomes: The number complexes observed within the PCP-SILAC interactome based on Markov clustering are shown 70%, 60% and 50% precision.

Figure S7, Fas-Mediated Changes In The Cytoplasmic And Membrane Interactomes At 1hour Post Treatment

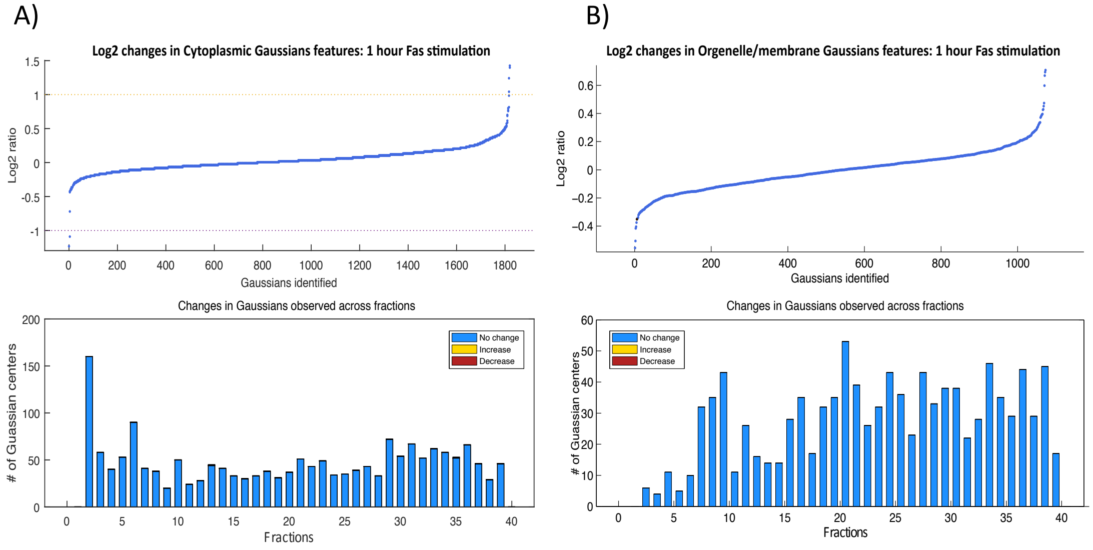

Figure S7, Fas-mediated changes in the cytoplasmic and membrane interactomes at 1hour post treatment: A) Observed changes in the cytosolic interactome in response to 1 h Fas-stimulation. In total 1820 Gaussian features were mapped across 40 fractions with few alterations within the interactome observed. **B)** Observed changes in the mitochondrial/membrane interactome in response to 1 h Fas-stimulation. In total, 1073 Gaussian features were mapped across 40 fractions with few alterations within the interactome observed.

Figure S8, tBID BN-PCP-SILAC Protein And Peptide Profiles

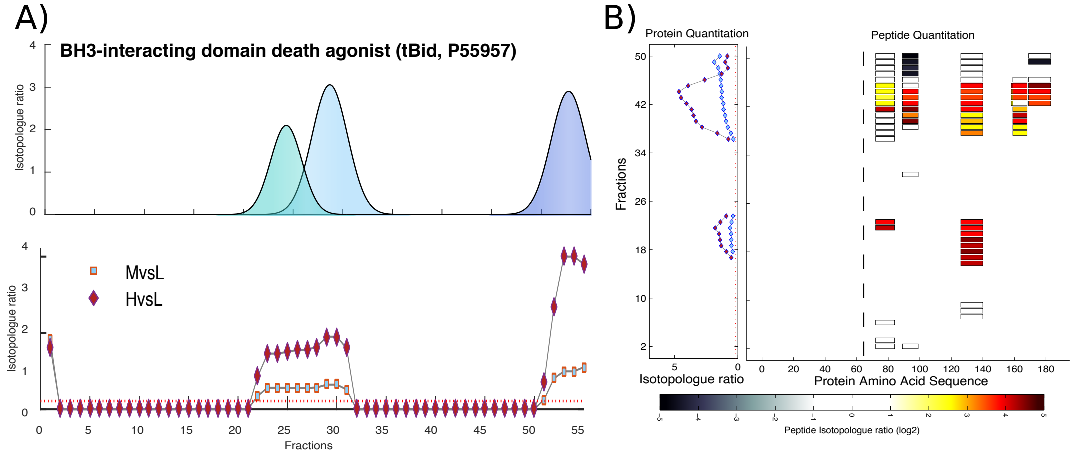

Figure S8, tBID BN-PCP-SILAC protein and peptide profiles: Protein and peptide profiles of tBID demonstrate that in response to apoptosis the level of tBID increase in the membrane. **A)** Protein profiles reveal multiple discrete regions within the BN gradient were tBID was observed, supporting that this protein form multiple membrane associations. **B)** Peptide profiles provide protein coverage information with the absent of N-terminal peptides supporting that the form of BID observed is tBID.

Figure S9, Examples Of Changes In The Nuclei Related Components Observed Within The Cytoplasmic Interactome

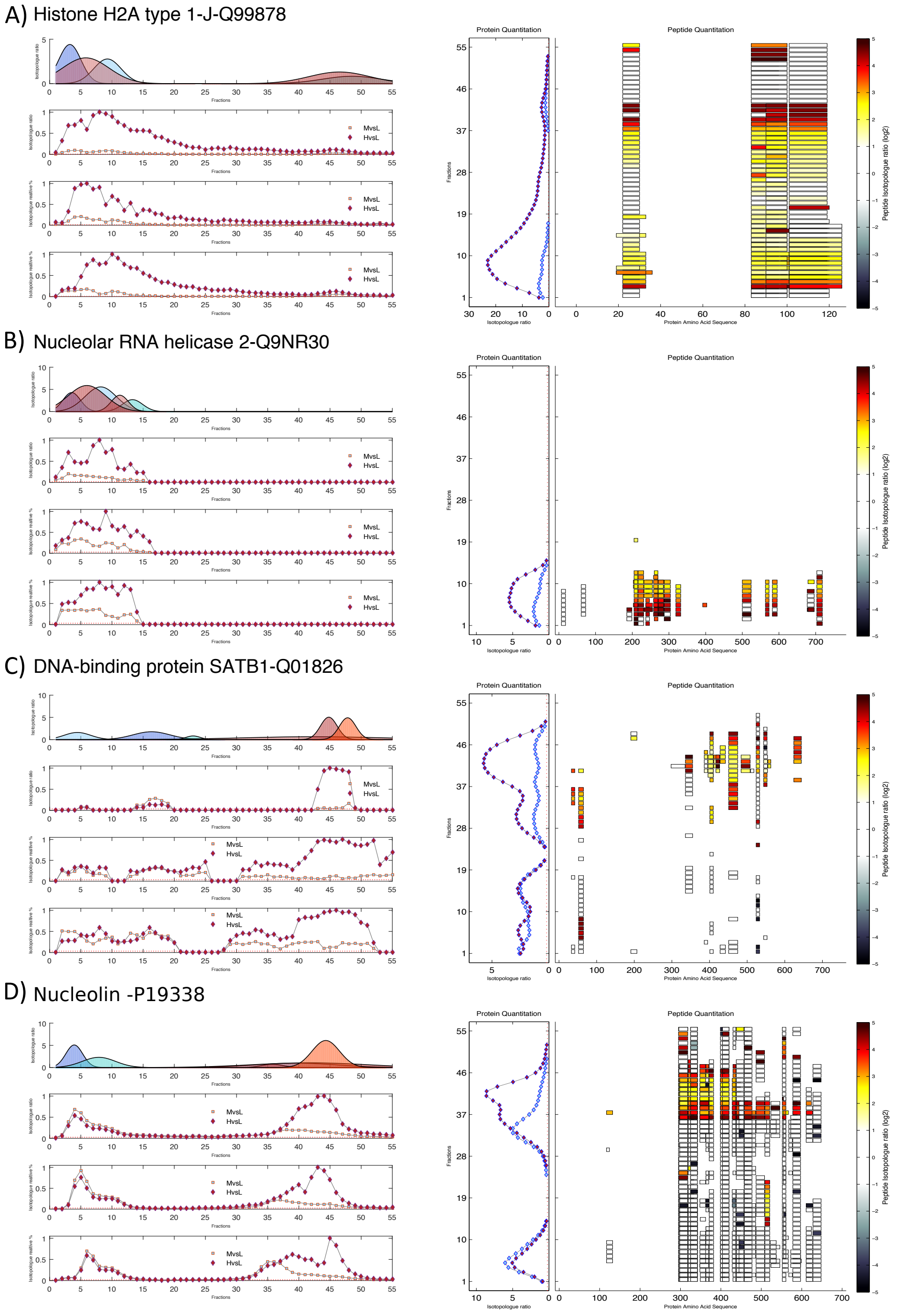

Figure S9, Changes in the protein and peptide profiles of nuclei related components observed in the cytoplasmic interactome: Consistent with the enrichment of protein-DNA complex subunit organization large changes in multiple nuclei related proteins were observed including **A)** Histone H2A 1-J (Q99878), **B)** Nucleolar RNA helicase 2 (Q9NR30), **C)** DNA-binding protein SATB1 (Q01826) and **D)** Nucleolin (P19338).

Figure S10, Analysis of Z-vad FMK stabilized N-termini.

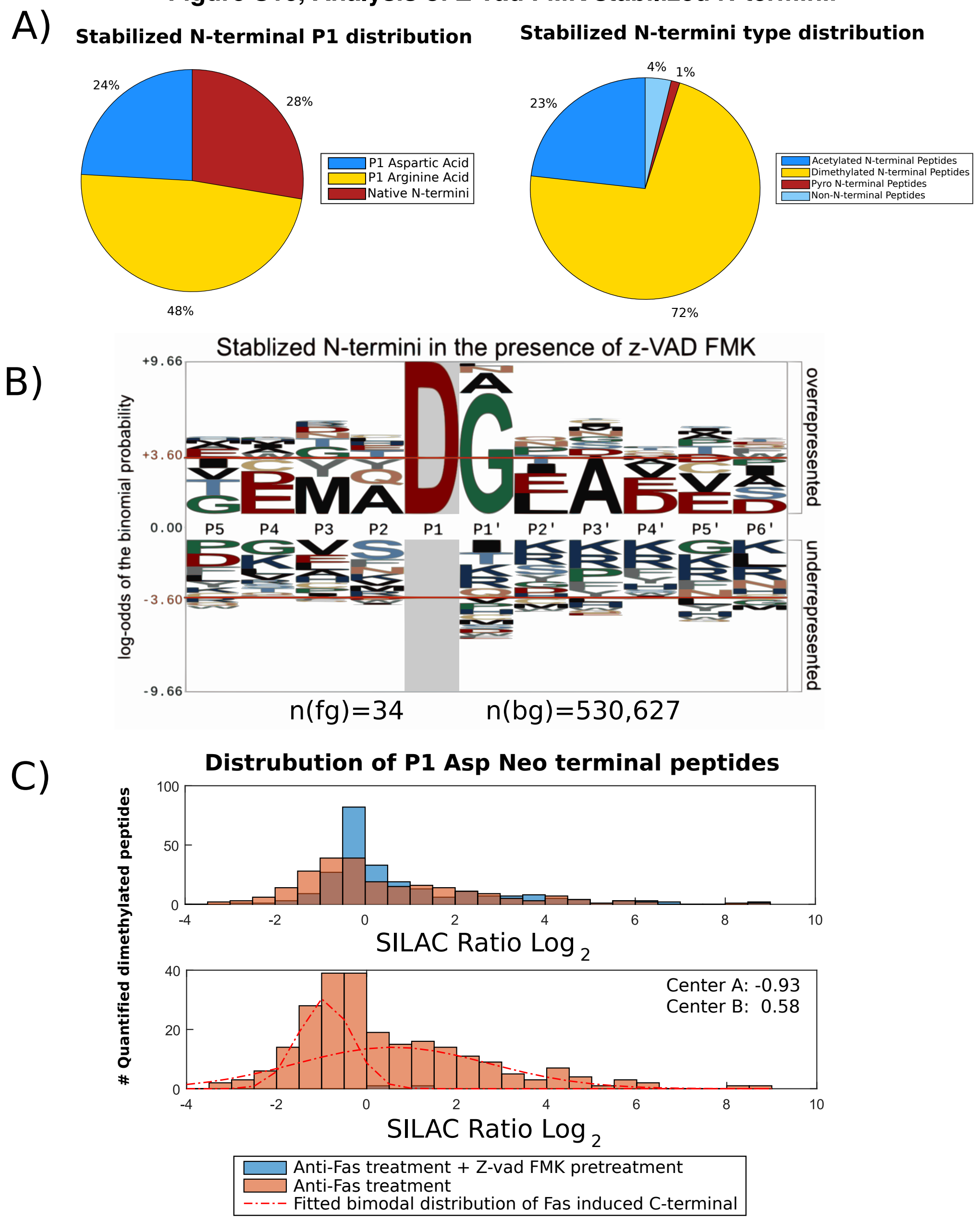

Figure S10, Analysis of Z-vad FMK stabilized N-termini. A) The distribution of observed P1 amino acid in stabilized N-termini are shown with native corresponding to all dimethylated peptides within five amino acid of the true N-termini. The Majority of stabilized N-termini correspond to dimethylated neo-termini consistent with the generation of cleavage events in response to Fas stimulation. **B)** Motif analysis of P1 aspartic acid neo-termini reveals a motif consistent with caspase cleavage specificity. **C)** Distribution of P1 aspartic acid neo-termini reveals in response to Fas stimulation without caspase inhibition. Without inhibition two populations of P1 aspartic acid neo termini can be seen, one that's decreases and is centered on -0.93 and one that increases centered on 0.53.

Figure S11, Alterative protease activity in response to Fas initiated apoptosis

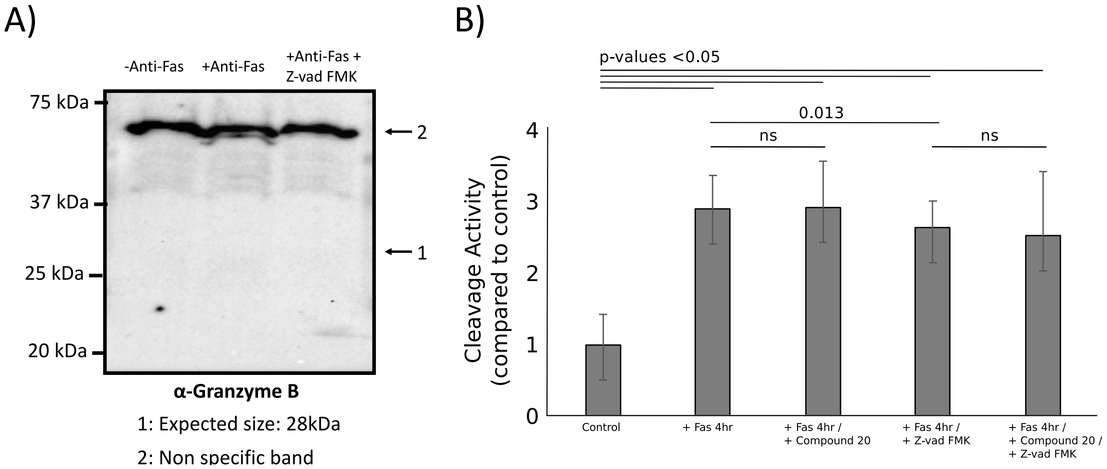

Figure S11, Investigation of alterative protease activities in response to Fas initiated apoptosis: A) Western blotting analysis of Granzyme B failed to detect an active form of Granzyme B in any of the condition examined in this study B). In vitro cleavage assays using Jurkat lysates and Ac-IEPD-pNA as a substrate show an increase in Asp-lytic activity in Fas treated cells. The observed lytic activity is not sensitive to the pre-incubation with a specific granzyme B Inhibitor Compound 20, suggesting that other (than Granzyme B) proteases are responsible for these cleavages.

Figure S12, Map of Filamin-B (O75369)

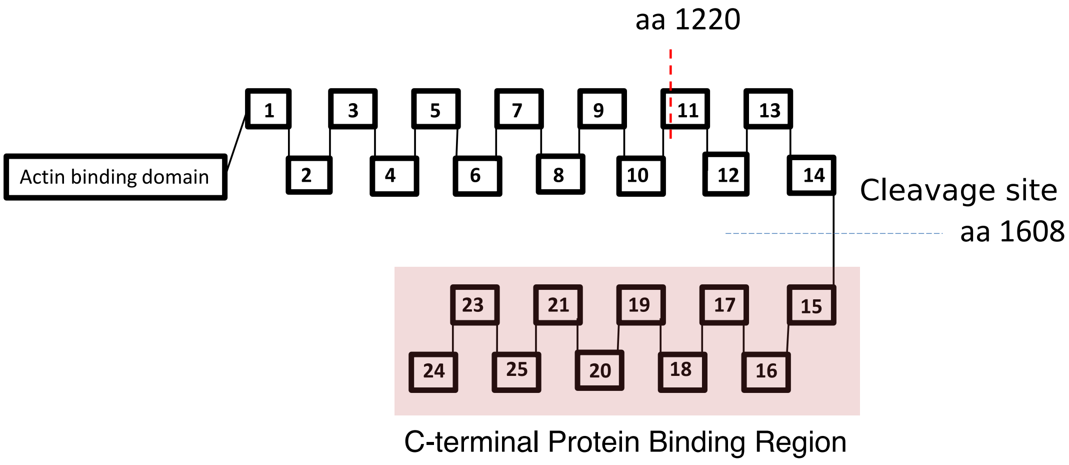

Figure S12, Map of Filamin-B (O75369): TAILS analysis reveals the present of a neo-N-terminus at position 1,220 generated in response to apoptosis. Uneveness analysis of the interactome data reveals a potential cleavage around positions 1600 which would separate the known C-terminal protein interaction binding domains from the N-terminal actin-binding domain.

Figure S13, Protein profile of known Caspase targets

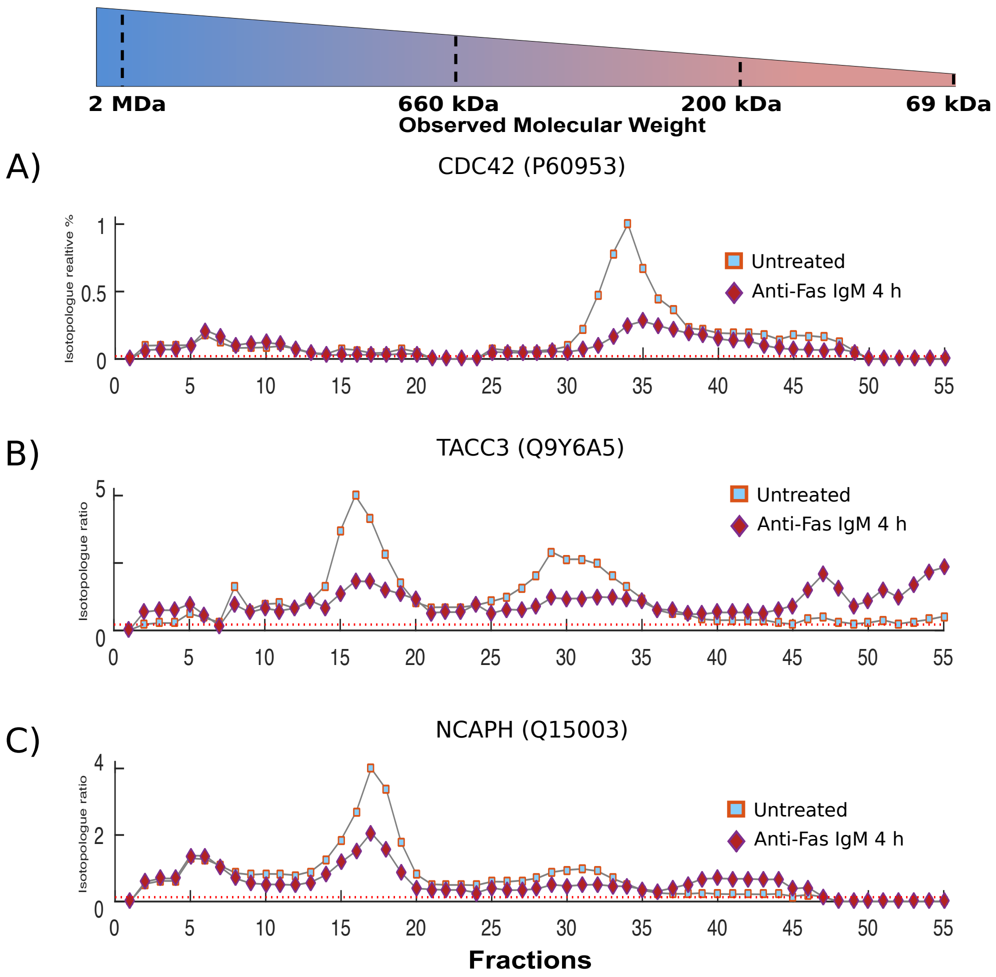

Figure S13, Investigation of protein specific proteolysis of known caspase targets: Protein profile for CDC-42 (P60953), **A)** TACC3 (Q9Y6A5), **B)** NCAPH (Q15003), **C)** reveals that in response to apoptosis the major Gaussian feature is lost.

Figure S14, NADH-ubiquinone oxidoreductase 75 kDa subunit

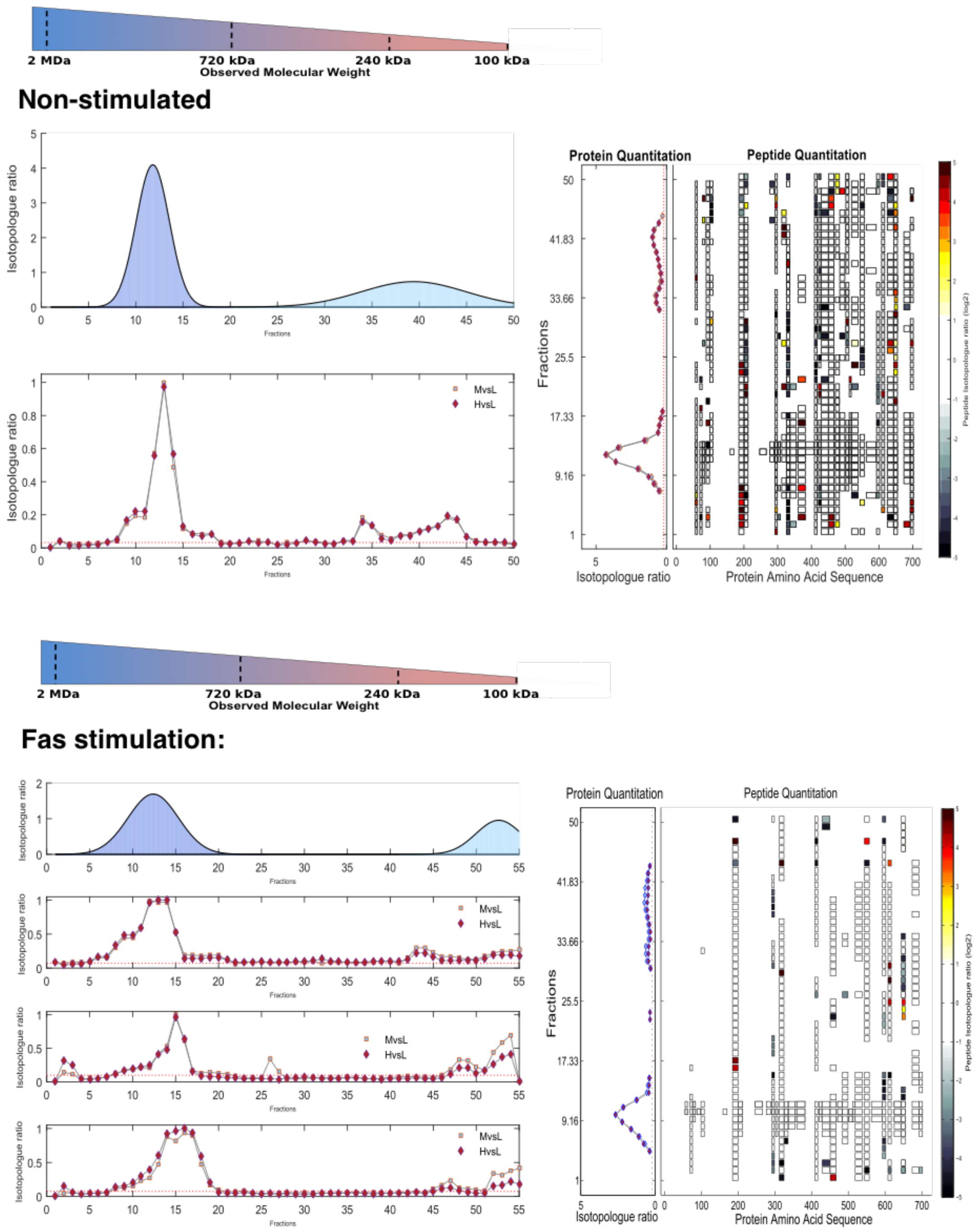

Figure S14, BN-PCP-SILAC Protein and Peptide Profiles of the known caspae-3 target NADH dehydrogenase (P28331): Within both non-treated and treated BN-PAGE protein and peptide profiles no changes in the associations are observed suggesting the known alteration leading to loss of mitochondrial transmembrane potential occur later in apoptosis progression.

Datasets descriptions

All MS date files generated for this manuscript are provide within the PRIDE repository accessible under the dataset identifier PXD00289. A description of each of the experiments are provided below and the associated files found within this repository are described within Table EV 25.

Experiment description

1) Membrane BN-PAGE PCP experiment (one dataset of 50 BN-PAGE fractions): Initial experiment to assess the feasibility and reproducibility of PCP-BN_PAGE. Three isotopically labelled populations of Jurkat cells (light, medium and heavy) were used to assess reproducibility by comparing the observed interactions and differences between medium and heavy profiles.
2) 4 hour Anti-Fas treatment cytoplasmic PCP SEC experiment (three datasets, each composed of 55 SEC fractions): Experiment designed to assess the effect of apoptosis on the soluble interactome. Three isotopically labelled populations of Jurkat cells (light, medium and heavy) were used, light cells were used as the reference population and added equally to all fractions; medium cells were untreated and heavy cells treated for four hours with Anti-Fas IgM.
3) 4 hour Anti-Fas treatment membrane PCP BN-PAGE experiment (three datasets, each composed of 55 BN-PAGE fractions): Experiment designed to assess the effect of apoptosis on the organelle/membrane interactome. Three isotopically labelled populations of Jurkat cells (light, medium and heavy) were used, light cells were used as the reference population and added equally to all fractions; medium cells were untreated and heavy cells treated for four hours with Anti-Fas IgM.
4) 1 hour Anti-Fas treatment cytoplasmic PCP SEC experiment (one dataset composed of 40 SEC fractions): Experiment designed to assess the effect of apoptosis on the soluble interactome. Three isotopically labelled populations of Jurkat cells (light, medium and heavy) were used, light cells were used as the reference population and added equally to all fractions; medium cells were untreated and heavy cells treated for one hour with Anti-Fas IgM.
5) 1 hour Anti-Fas treatment membrane PCP BN-PAGE experiment (one dataset composed of 40 BN-PAGE fractions): Experiment designed to assess the effect of apoptosis on the organelle/membrane interactome. Three isotopically labelled populations of Jurkat cells (light, medium and heavy) were used, light cells were used as

the reference population and added equally to all fractions; medium cells were untreated and heavy cells treated for one hour with Anti-Fas IgM.

6) N-tail enrichment experiments (three biological replicates): Experiment designed to assess the N-termini of proteins within protein complexes after 4 hour anti-Fas treatment in the presence of absence of a pan caspase inhibitor. Three isotopically labelled populations of Jurkat cells (light, medium and heavy) were used, light cells were used as the reference population and added equally to all fractions; medium cells were untreated and heavy cells treated for four hours with Anti-Fas IgM.

References

Babu M, Vlasblom J, Pu S, Guo X, Graham C, Bean BD, Burston HE, Vizeacoumar FJ, Snider J, Phanse S, Fong V, Tam YY, Davey M, Hnatshak O, Bajaj N, Chandran S, Punna T, Christopolous C, Wong V, Yu A et al (2012) Interaction landscape of membrane-protein complexes in Saccharomyces cerevisiae. *Nature* **489:** 585-589

Bent SJ, Forney LJ (2008) The tragedy of the uncommon: understanding limitations in the analysis of microbial diversity. *ISME J* **2:** 689-695

Chambers MC, Maclean B, Burke R, Amodei D, Ruderman DL, Neumann S, Gatto L, Fischer B, Pratt B, Egertson J, Hoff K, Kessner D, Tasman N, Shulman N, Frewen B, Baker TA, Brusniak MY, Paulse C, Creasy D, Flashner L et al (2012) A cross-platform toolkit for mass spectrometry and proteomics. *Nat Biotechnol* **30:** 918-920

Cox J, Mann M (2008) MaxQuant enables high peptide identification rates, individualized p.p.b.-range mass accuracies and proteome-wide protein quantification. *Nature biotechnology* **26:** 1367-1372

Enright AJ, Van Dongen S, Ouzounis CA (2002) An efficient algorithm for large-scale detection of protein families. *Nucleic Acids Res* **30:** 1575-1584

Frezza C, Cipolat S, Scorrano L (2007) Organelle isolation: functional mitochondria from mouse liver, muscle and cultured fibroblasts. *Nat Protoc* **2:** 287-295

Guruharsha KG, Rual JF, Zhai B, Mintseris J, Vaidya P, Vaidya N, Beekman C, Wong C, Rhee DY, Cenaj O, McKillip E, Shah S, Stapleton M, Wan KH, Yu C, Parsa B, Carlson JW, Chen X, Kapadia B, VijayRaghavan K et al (2011) A protein complex network of Drosophila melanogaster. *Cell* **147:** 690-703

Heide H, Bleier L, Steger M, Ackermann J, Drose S, Schwamb B, Zornig M, Reichert AS, Koch I, Wittig I, Brandt U (2012) Complexome Profiling Identifies TMEM126B as a Component of the Mitochondrial Complex I Assembly Complex. *Cell metabolism* **16:** 538-549

Kleifeld O, Doucet A, auf dem Keller U, Prudova A, Schilling O, Kainthan RK, Starr AE, Foster LJ, Kizhakkedathu JN, Overall CM (2010) Isotopic labeling of terminal amines in complex samples identifies protein N-termini and protease cleavage products. *Nat Biotechnol* **28:** 281-288

Kleifeld O, Doucet A, Prudova A, auf dem Keller U, Gioia M, Kizhakkedathu JN, Overall CM (2011) Identifying and quantifying proteolytic events and the natural N terminome by terminal amine isotopic labeling of substrates. *Nat Protoc* **6:** 1578-1611

Kristensen AR, Gsponer J, Foster LJ (2012) A high-throughput approach for measuring temporal changes in the interactome. *Nat Methods*

Li H, Zhu H, Xu CJ, Yuan J (1998) Cleavage of BID by caspase 8 mediates the mitochondrial damage in the Fas pathway of apoptosis. *Cell* **94:** 491-501

O'Shea JP, Chou MF, Quader SA, Ryan JK, Church GM, Schwartz D (2013) pLogo: a probabilistic approach to visualizing sequence motifs. *Nat Methods* **10:** 1211-1212

Rappsilber J, Ishihama Y, Mann M (2003) Stop and go extraction tips for matrix-assisted laser desorption/ionization, nanoelectrospray, and LC/MS sample pretreatment in proteomics. *Anal Chem* **75:** 663-670

Rappsilber J, Mann M, Ishihama Y (2007) Protocol for micro-purification, enrichment, pre-fractionation and storage of peptides for proteomics using StageTips. *Nature protocols* **2:** 1896-1906

Rogers LD, Fang Y, Foster LJ (2010) An integrated global strategy for cell lysis, fractionation, enrichment and mass spectrometric analysis of phosphorylated peptides. *Molecular bioSystems* **6:** 822-829

Ruepp A, Waegele B, Lechner M, Brauner B, Dunger-Kaltenbach I, Fobo G, Frishman G, Montrone C, Mewes HW (2010) CORUM: the comprehensive resource of mammalian protein complexes--2009. *Nucleic Acids Res* **38:** D497-501

Scaffidi C, Fulda S, Srinivasan A, Friesen C, Li F, Tomaselli KJ, Debatin KM, Krammer PH, Peter ME (1998) Two CD95 (APO-1/Fas) signaling pathways. *EMBO J* **17:** 1675-1687

Schaab C, Geiger T, Stoehr G, Cox J, Mann M (2012) Analysis of high accuracy, quantitative proteomics data in the MaxQB database. *Molecular & cellular proteomics : MCP* **11:** M111 014068

Schilling B, Rardin MJ, MacLean BX, Zawadzka AM, Frewen BE, Cusack MP, Sorensen DJ, Bereman MS, Jing E, Wu CC, Verdin E, Kahn CR, Maccoss MJ, Gibson BW (2012) Platform-independent and label-free quantitation of proteomic data using MS1 extracted ion chromatograms in skyline: application to protein acetylation and phosphorylation. *Mol Cell Proteomics* **11:** 202-214

Scott NE, Brown LM, Kristensen AR, Foster LJ (2015) Development of a computational framework for the analysis of protein correlation profiling and spatial proteomics experiments. *J Proteomics* **118:** 112-129

Shannon CE, Weaver W (1949) *The Mathematical Theory of Communication*, Urbana, IL: University of Illinois Press.

Shevchenko A, Tomas H, Havlis J, Olsen JV, Mann M (2006) In-gel digestion for mass spectrometric characterization of proteins and proteomes. *Nature protocols* **1:** 2856-2860

Stoehr G, Schaab C, Graumann J, Mann M (2013) A SILAC-based approach identifies substrates of caspase-dependent cleavage upon TRAIL-induced apoptosis. *Mol Cell Proteomics*

Van Damme P, Martens L, Van Damme J, Hugelier K, Staes A, Vandekerckhove J, Gevaert K (2005) Caspase-specific and nonspecific in vivo protein processing during Fas-induced apoptosis. *Nat Methods* **2:** 771-777

Vizcaino JA, Deutsch EW, Wang R, Csordas A, Reisinger F, Rios D, Dianes JA, Sun Z, Farrah T, Bandeira N, Binz PA, Xenarios I, Eisenacher M, Mayer G, Gatto L, Campos A, Chalkley RJ, Kraus HJ, Albar JP, Martinez-Bartolome S et al (2014) ProteomeXchange provides globally coordinated proteomics data submission and dissemination. *Nat Biotechnol* **32:** 223-226

Weis M, Schlegel J, Kass GE, Holmstrom TH, Peters I, Eriksson J, Orrenius S, Chow SC (1995) Cellular events in Fas/APO-1-mediated apoptosis in JURKAT T lymphocytes. *Exp Cell Res* **219:** 699-708

Wittig I, Braun HP, Schagger H (2006) Blue native PAGE. *Nat Protoc* **1:** 418-428
