# Supplementary material for: Interactome disassembly during apoptosis occurs independent of caspase cleavage
Source: Mol Syst Biol. 2017 Jan 12;13(1):906. doi: 10.15252/msb.20167067 (PMC5293159; doi:10.15252/msb.20167067)
Supplement: Supplementary file 2 — Expanded View Figures PDF [file MSB-13-906-s002.pdf]

Expanded View Figures

Figure EV1. Diagrammatic workflow of study design.

A, B This study utilizes two proteomic strategies to explore the effect of early Fas-mediated apoptosis on cells: (A) an interactome approach and (B) an N-terminal approach. Within the interactome workflow (A), protein complexes were isolated and separated using either SEC, depictured as the column for cytoplasmic complexes or BN-PAGE, depicted as the gel for membrane complexes. Within the protein N-termini workflow (B), protein complexes were isolated and subjected to N-terminal enrichment analysis (N-TAILS). Within both workflows, three SILAC-labeled cell populations of Jurkat cells were grown for five passages prior to the study. (A) Interactome workflow: For the analysis of the interactome, heavy-labeled cells (labeled with Lys-8 and Arg-10) were treated with 250 ng/ml of anti-Fas IgM (CH11, EMD Millipore) for 4 h, while medium (labeled with Lys-4 and Arg-6) and light (labeled with Lys-0 and Arg-0) cells were left untreated. Protein complexes from the cytosol and organelles were isolated and separated with SEC or BN-PAGE, denoted respectively. The resulting samples were digested, analyzed by LC-MS/MS, and processed using MATLAB to generate the resulting interaction networks. (B) N-terminal enrichment workflow: For the analysis of the N-termini, heavy-labeled cell were treated with 250 ng/ml of anti-Fas IgM for 4 h after an initial 1-h treatment with 20 μM Z-vad-FMK. Medium-labeled cells were treated with 250 ng/ml of anti-Fas IgM for 4 h, and light-labeled cells were left untreated. Protein complexes from the cytosol were isolated and subjected to N-terminal enrichment followed by analysis using LC-MS/MS.

© 2017 The Authors

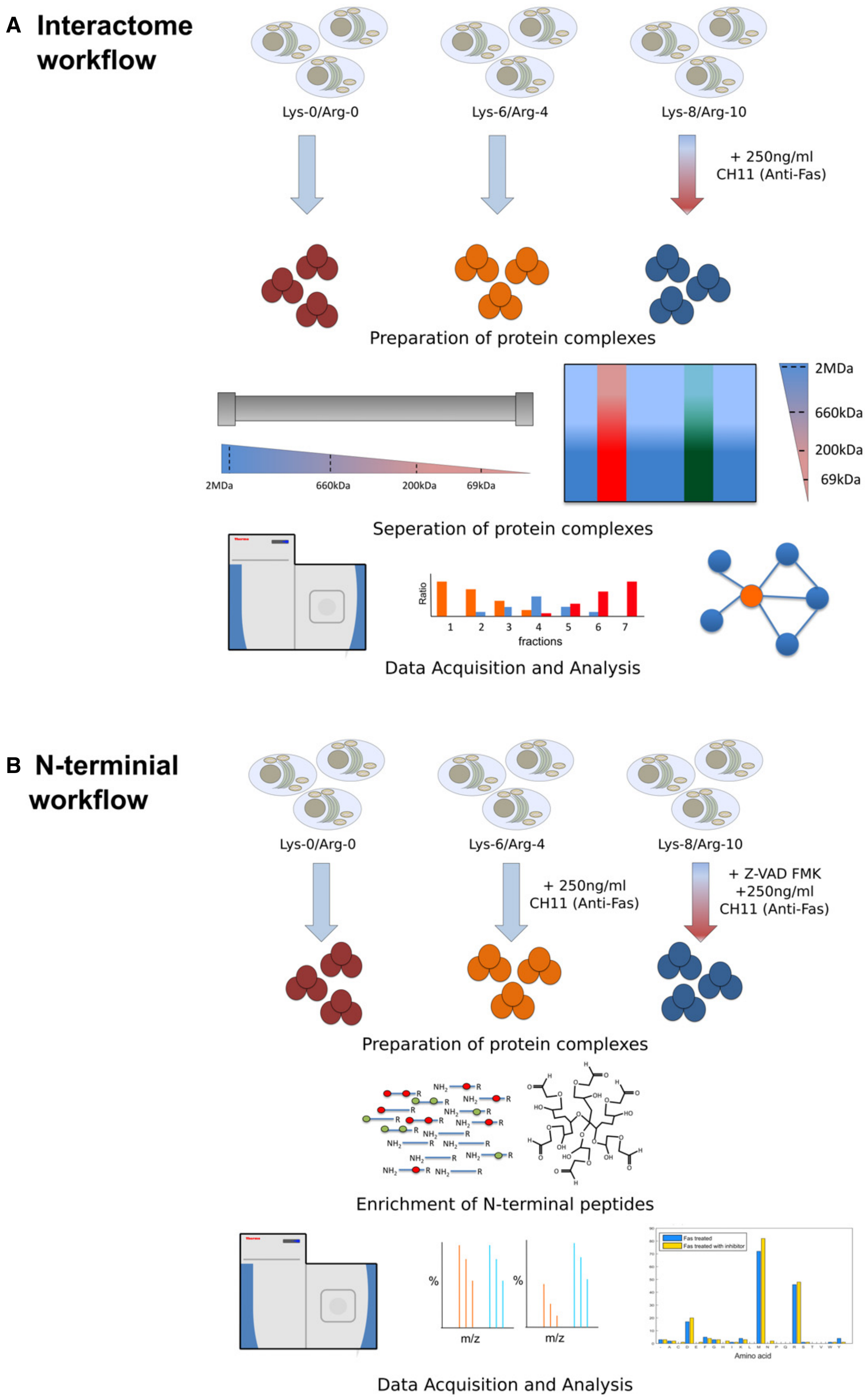

Figure EV1.

© 2017 The Authors

Figure EV2. Interaction properties of PCP-BN-SILAC.

A Vertex degree distribution of interactions (determined at 70% precision), the vertex degree distribution of the connectivity of proteins within the PCP-BN-SILAC interactome demonstrates a power law fit. The dashed line represents the best-fit power law (r^2 of 0.906) corresponding to the equation $y = -1.249x^{0.445}$.

B Precision-recall properties of PCP-BN-SILAC. The precision-recall properties of the Euclidean Distance and the Fitted Gaussian features were determined using the CORUM database. Both Euclidean Distance and the Fitted Gaussian features of either isotopologue channel demonstrated robust discriminatory power with greater similarity of these features being associated with a higher level of precision.

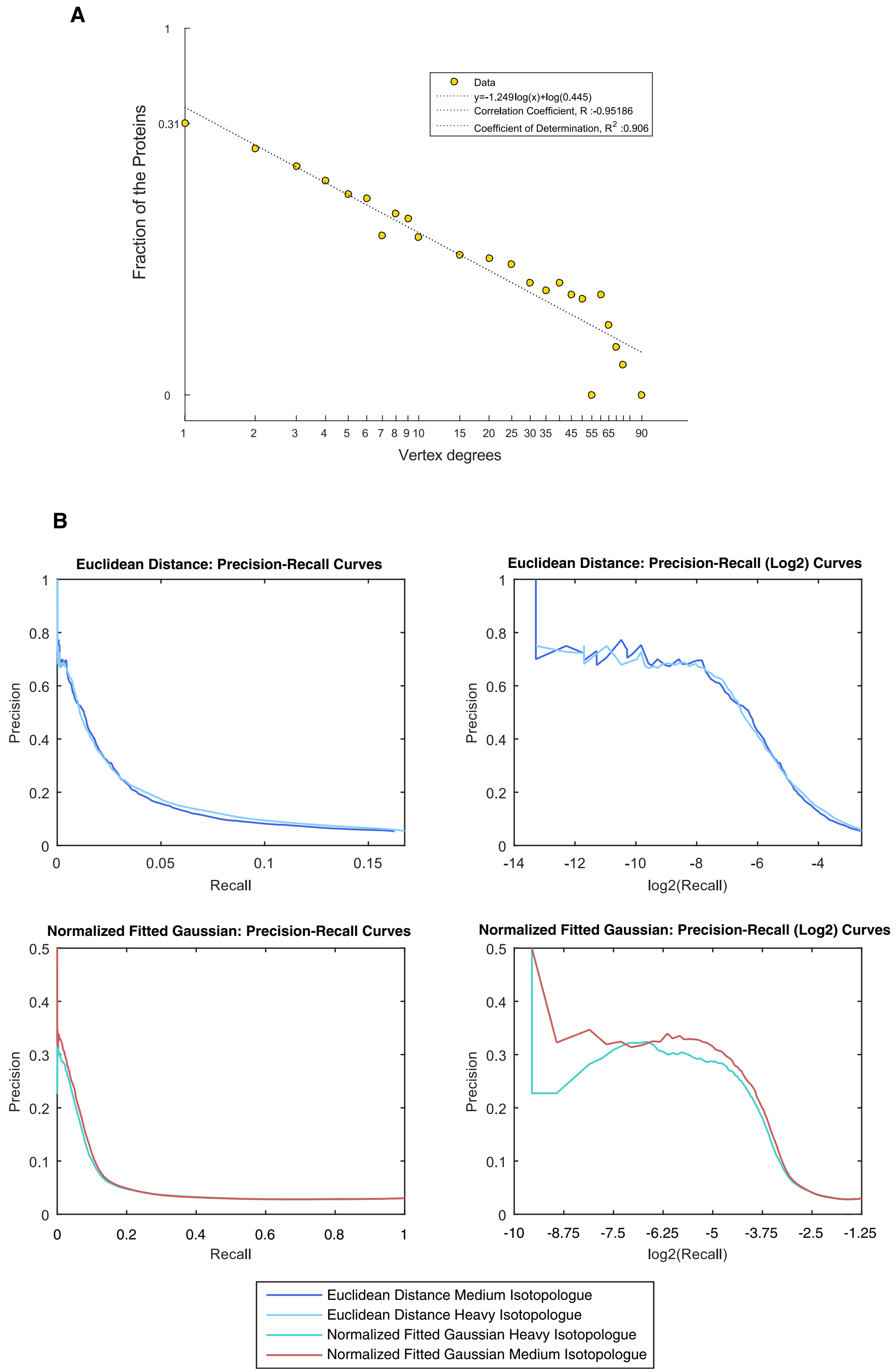

Figure EV2.

© 2017 The Authors

Figure EV3. Condensin I complex members' peptide maps.
The protein and peptide maps for all five members (NCAPH, NCAPD2, NCAPG, Structural maintenance of chromosomes protein 2, and Structural maintenance of chromosomes protein 4) of condensin I are shown. Consistent with changes observed at the protein level within the five condensin I members, the initiation of apoptosis leads to a decrease in the detected peptide levels observed across the whole SEC gradient.

© 2017 The Authors

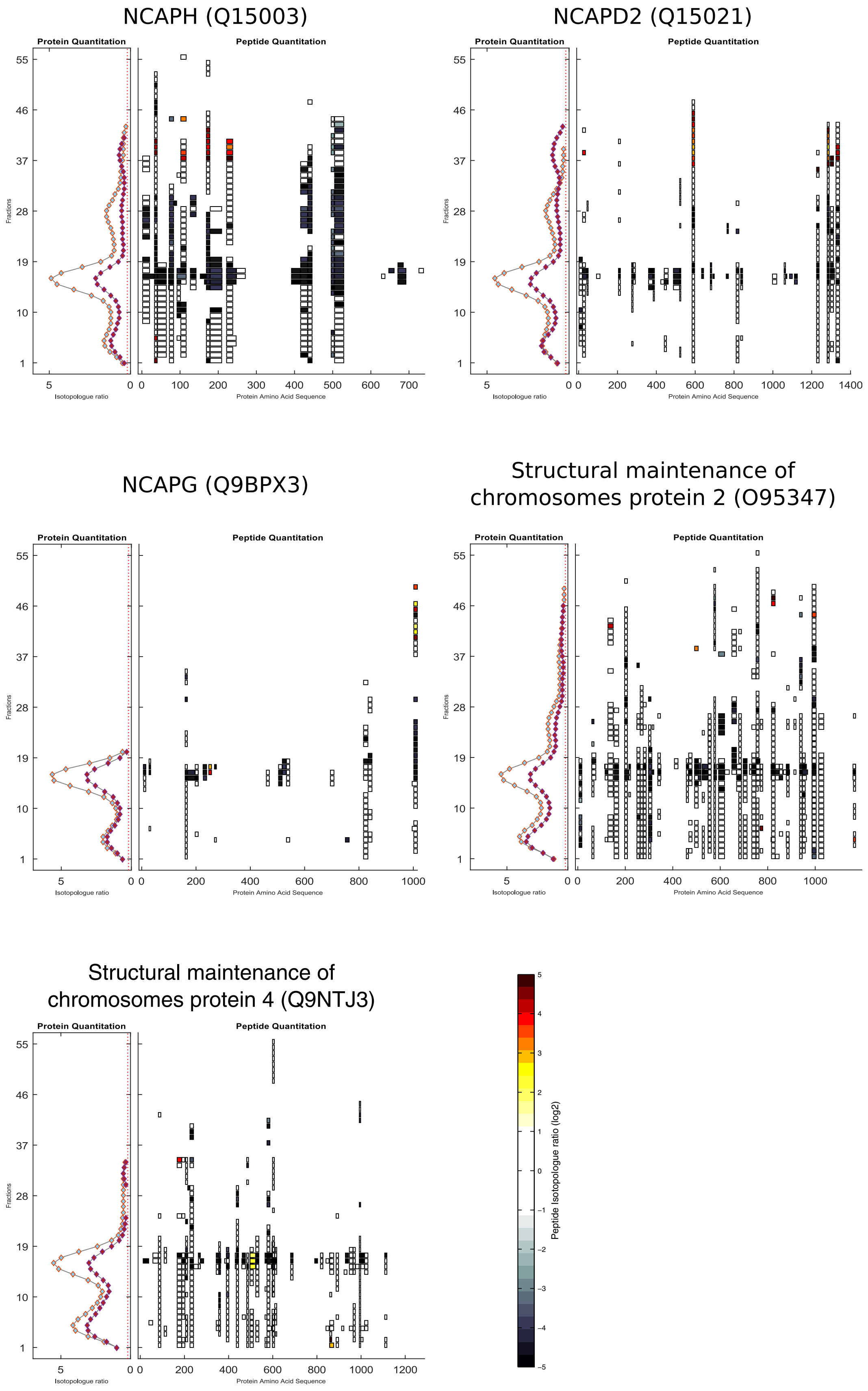

Figure EV3.

© 2017 The Authors

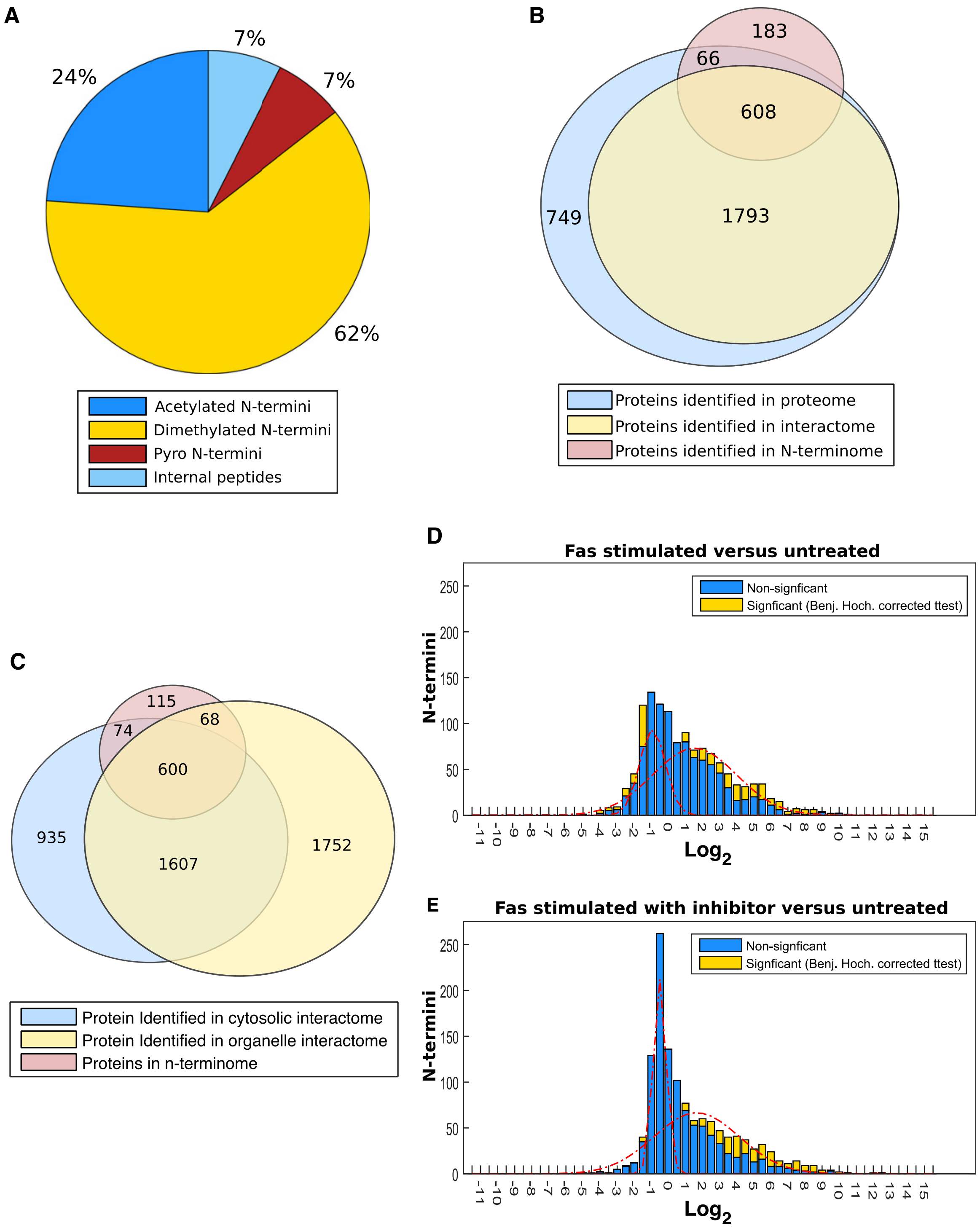

Figure EV4. Analysis and comparison of the observed protein N-termini of Fas-induced apoptosis to the interactome.

A Analysis of identified peptide shows 86% of all peptides were N-terminal in origins.

B Overlap of N-terminome with observed interactome, 608 proteins out of the 857 observed within the N-terminome were observed in the interactome with a further 66 identified within the interactome with no Gaussian features fitted.

C Overlap of proteins identified in the N-terminome, cytosolic interactomes, and organelle interactomes.

D, E Histogram distribution of the ratio of observed N-termini peptide of Fas treatment (D) and Fas treatment with the caspase inhibitor Z-vad-FMK (E) compared to untreated cells. N-termini which were determined to be statistically significant by two-sided *t*-test with Benjamini–Hochberg correction are shown in yellow. Fitted Gaussians corresponding to the modeled observed distributions are shown in red.

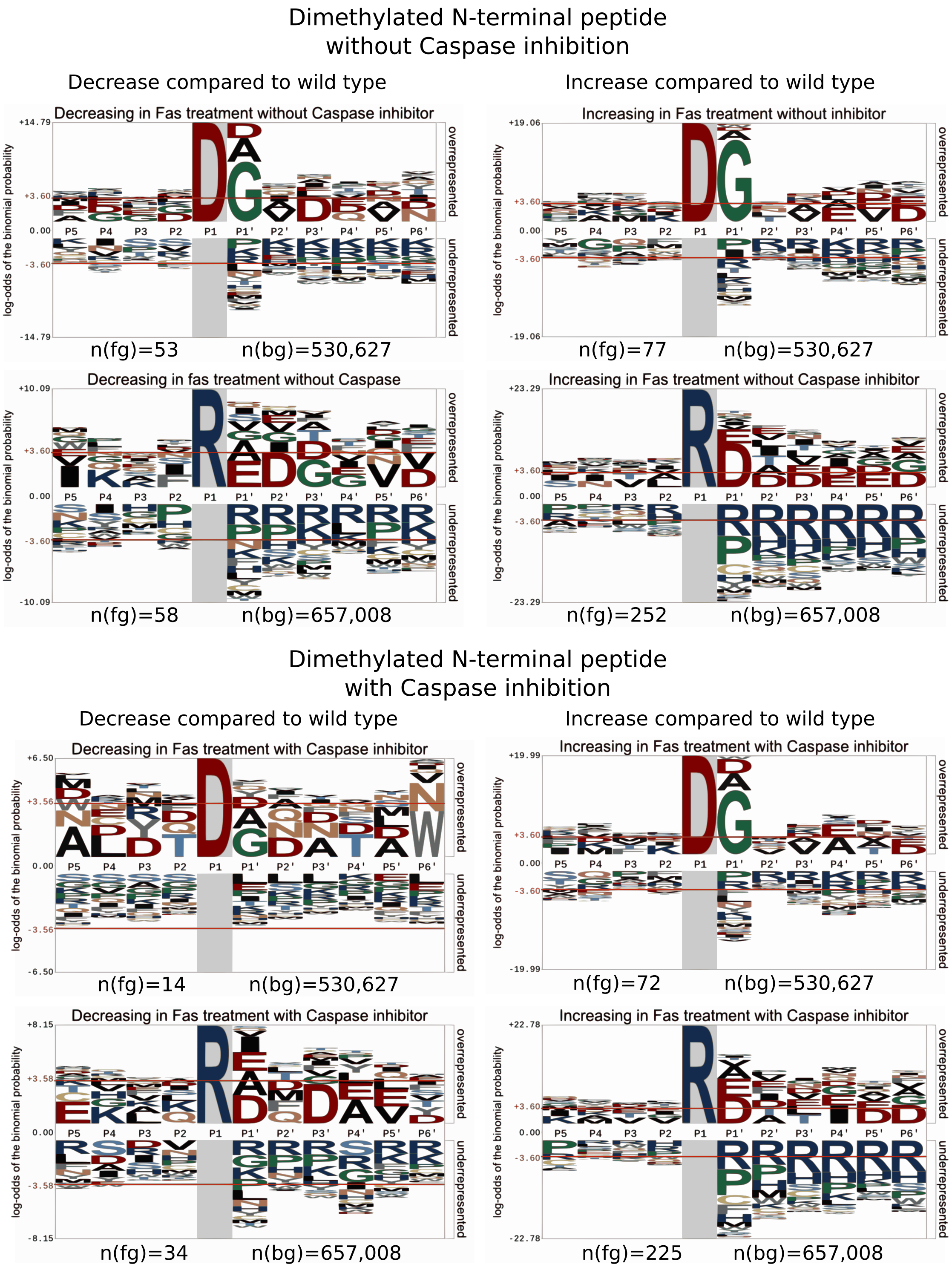

Figure EV5. Cleavage motif analysis of dimethylated N-terminal peptides with D or R in the P1 positions.

Visualization of the P5–P6′ amino acid sequences of dimethylated N-terminal peptides alignments observed to change in response to Fas stimulation. Peptides were aligned based on share P1 residues and direction of change and assessed against the human proteome. For peptide with an aspartic acid in the P1 position which decrease in response to apoptosis without caspase inhibition, a clear caspase-2/3/7 motif of D-E-X-D|G (Wejda *et al*, 2012) was observed. This motif was absent in peptides with an aspartic acid in the P1 position, which decreases in response to apoptosis with caspase inhibition. In contrast, peptide with an aspartic acid in the P1 position, which increase in response to apoptosis, showed a D|G preference, yet no enrichment in acidic residues was observed in the P4-2 positions. Within peptide with an arginine in the P1 position regardless of direction in the change peptide showed an enrichment in acidic residues at the P′1 position. For all visualizations, the number of peptides used for alignment, *n*(fg), and the number of peptides used within the background reference proteome, *n*(bg), are provided.

© 2017 The Authors
