# Supplementary material for: Interactome disassembly during apoptosis occurs independent of caspase cleavage
Source: Mol Syst Biol. 2017 Jan 12;13(1):906. doi: 10.15252/msb.20167067 (PMC5293159; doi:10.15252/msb.20167067)
Supplement: Supplementary file 27 — Code EV1 [file MSB-13-906-s027.zip › Script_descriptions.docx]

**MATLAB based analysis:** All experiments were processed using the bioinformatics approach outlined within (Scott et al, 2015). In addition to the Gaus.m, Alignment.m, Comparison.m, PPI.m scripts described previously, additional scripts membrane.m, peptide_mapping.m and cluster.m were created to further enhance the analysis and visualization of data. The precision of protein interaction networks generated during analysis were calculated at two levels, at the local level; corresponding to all interactions within an isotopologue channel of a single experiment, and at the global level; corresponding to the combined precision of all interaction across all isotopologue channels and replicates. The desired precisions of 70%, 60% and 50% were generated by optimisation of the parameters required to reach these precisions threshold at the local level within a given isotopologue channel and then combining the determined interactions into a single networks. Membrane.m enables GO terms possessed by assigned interactions to be assessed based on the percentage of membrane associated GO terms and matching terms. Peptide_mapping.m enables the analysis and visualization of peptide information for individual protein groups within a PCP experiment, similarly to a previous report (Stoehr et al, 2013). Clustering was accomplished using a Markov clustering approach (Babu et al, 2012; Enright et al, 2002; Guruharsha et al, 2011). Peptide quantitative information is utilized to assess evenness and diversity using a Shannon index (Bent & Forney, 2008; Shannon & Weaver, 1949). All the scripts described, together with representative test datasets, are available from our website (http://www.chibi.ubc.ca/faculty/leonard-foster/foster-lab/softwares/). A complete list of the scripted used within this manuscript is provide below.

**Complete description of scripts and dependences**

| **Script (File name and dependences)** | **Folder** | **Function (Figure number where output is shown)** |
| --- | --- | --- |
| Gaus.m (Gaus_build_24_1.m) | 1_Gaussian processing | Guassian Fitting of Data |
| Alignment.m (Alignment_Version_4_3.m) | 2_Alignment processing | Alignment of Biological replicate |
| Comparison.m (Comparison.m, dependences: mwwtest.m) | 3_comparesion | Enable the comparison of observed interactomes |
| ROC.m (ROC.m, dependences: myWhiten.m, myPCA.m, myCenter.m, d2p.m, strjoin.m, tsne.m, tsne_d.m, tsne_p.m, required) | 4_ROC | The assignment of PPI from PCP datasets |
| peptide_mapping.m (Mapping_peptide.m) | 5_peptide_mapping | Visualization of peptide data observed across PCP experiments |
| Clustering.m (Clustering.m, dependences: mcl.m , mcl_two.m, strjoin.m) | 6_Clustering_complexes | Markov clustering of PPI assignments |
| Complex_precision_v1.m (Complex_precision_v1.m, dependence: strjoin.m) | 7_Precision_complexs | Visualization of PPI at different levels of precision (See Figure 2C ,D and S6)* |
| Degree_distrubation.m (Degree_distrubation_Cytoplasmic_membrane_interactomes.m, dependences: strjoin.m) | 8_Degree_distribution | Visualization of network connectivity(See Figure EV2A and S5)* |
| Venn_diagram_overlap_between_BNE.m (Venn_diagram_overlap_between_BNE.m, dependences: venn.m) | 9_Venn_diagram_showing overlap | Visualization of overlap between observed mitochondrial proteome and previous studies (see Figure S2B)* |
| Membrane.m (membrane_interaction_script_version6.m, dependences: strjoin.m) | 10_Membrane assignments | Visualization of membrane assignments (See figure 1B)* |
| Tails_analysis.m (PD_tails_analysis_Skyline_Scaffold23_MWW_BH.m, dependences: mwwtest.m, strjoin.m, venn.m) | 11_Combined TAILS data | Visualization and analysis of N-TAILS datasets (See figure 5 and EV4)* |
| Comparsion_TAILS_to_interactome.m (dependences: venn.m) | 12_Comparsion_TAILS_to_interactome | Visualization and analysis comparing the N-termini data to the Interactome (See figure 5)* |

*Provided for transparency of analysis and visualization
